# *Candida antarctica* Lipase A-Based Enantiorecognition of a Highly Strained 4-Dibenzocyclooctynol (DIBO) Used for PET Imaging

**DOI:** 10.3390/molecules25040879

**Published:** 2020-02-17

**Authors:** Saija Sirén, Käthe M. Dahlström, Rakesh Puttreddy, Kari Rissanen, Tiina A. Salminen, Mika Scheinin, Xiang-Guo Li, Arto Liljeblad

**Affiliations:** 1 Laboratory of Synthetic Drug Chemistry, Institute of Biomedicine, University of Turku, Kiinamyllynkatu 10, FI-20520 Turku, Finland; saija.siren@utu.fi; 2 Biochemistry, Faculty of Science and Engineering, Åbo Akademi University, Tykistökatu 6 A, FI-20520 Turku, Finland; 3 Structural Bioinformatics Laboratory, Biochemistry, Faculty of Science and Engineering, Åbo Akademi University, Tykistökatu 6 A, FI-20520 Turku, Finland; Kathe.Dahlstrom@abo.fi (K.M.D.); Tiina.Salminen@abo.fi (T.A.S.); 4 Department of Chemistry, University of Jyväskylä, P.O. Box 35, FI-40014 Jyväskylä, Finlandkari.t.rissanen@jyu.fi (K.R.); 5 Institute of Biomedicine, University of Turku, and Unit of Clinical Pharmacology, Turku University Hospital, FI-20521 Turku, Finland; mschein@utu.fi; 6 Turku PET Centre, Åbo Akademi University and University of Turku, Kiinamyllynkatu 4-8, FI-20521 Turku, Finland; 7 Turku PET Centre, Turku University Hospital, Kiinamyllynkatu 4-8, FI-20521 Turku, Finland

**Keywords:** biocatalysis, lipase A from *Candida antarctica*, DIBO, kinetic resolution, molecular modeling

## Abstract

The enantiomers of aromatic 4-dibenzocyclooctynol (DIBO), used for radiolabeling and subsequent conjugation of biomolecules to form radioligands for positron emission tomography (PET), were separated by kinetic resolution using lipase A from *Candida antarctica* (CAL-A). In optimized conditions, (*R*)-DIBO [(*R*)-**1**, ee 95%] and its acetylated (*S*)-ester [(*S*)-**2**, ee 96%] were isolated. In silico docking results explained the ability of CAL-A to differentiate the enantiomers of DIBO and to accommodate various acyl donors. Anhydrous MgCl_2_ was used for binding water from the reaction medium and, thus, for obtaining higher conversion by preventing hydrolysis of the product (*S*)-**2** into the starting material. Since the presence of hydrated MgCl_2_·6H_2_O also allowed high conversion or effect on enantioselectivity, Mg^2+^ ion was suspected to interact with the enzyme. Binding site predictions indicated at least two sites of interest; one in the lid domain at the bottom of the acyl binding pocket and another at the interface of the hydrolase and flap domains, just above the active site.

## 1. Introduction

**Scheme 1:**
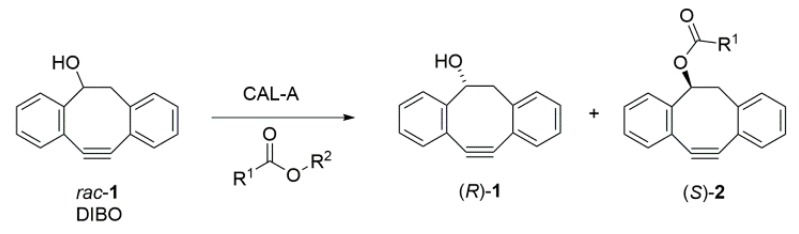
*Candida antarctica* (CAL-A)-catalyzed kinetic resolution of 4-dibenzocyclooctynol (DIBO).

Positron emission tomography (PET) imaging provides a method for observing metabolic processes and biological macromolecules in living organisms and, accordingly, an opportunity to diagnose various human diseases. To provide efficient and specific target binding, oligonucleotides, peptides, or antibodies are often coupled to radionuclides with a linker to form radiopharmaceuticals [1,2]. 11,12-Didehydro-5,6-dihydrodibenzo[a,e]cyclooctyn-5-ol (DIBO, Scheme 1), most commonly called 4-dibenzocyclooctynol in the literature, is used for conjugation of biomolecules and radiolabels (Scheme 1) [3]. Racemic DIBO has previously been used as a component of an antibody-based imaging agent that targets a biomarker of pancreatic cancer, CA19.9 [4]. Similarly, to non-radioactive pharmaceuticals, high enantiopurity is desirable in radiopharmaceuticals since enantiopure compounds typically improve safety and efficacy [5]. DIBO, however, poses a challenge for any asymmetric catalyst, since it contains two similar-sized bulky structural moieties adjacent to its asymmetric center, making its enantiomeric recognition very difficult. Furthermore, two fused benzene rings with a cyclooctyne ring makes DIBO highly strained and, therefore, the enantiomers of DIBO have so far remained unseparated. Among the few known enzymes acting on this type of substrates, lipase A from *Candida antarctica* (CAL-A) is known to accept bulky secondary alcohols [6,7] and similar amines [8,9].

CAL-A is structurally unique, representing a novel structural family of lipases [10]. It has a catalytic triad consisting of three amino acid residues, S184, D334, and H366, a well-defined lid domain (residues 217–308) as well as a classical α/β-hydrolase domain. In addition to the lid, CAL-A has a separate flap domain (residues 425–441) surrounding the nucleophile binding site. [11] During interfacial activation, the flap swings out, exposing the active site. As a result, the available space on the nucleophile binding site becomes practically infinite, which explains the ability of CAL-A to accept sterically hindered substrates, even tertiary alcohols [12,13] and secondary amines [14,15], i.e., substrates that are seldom accepted by other lipases. 

We investigated the ability of lipases and especially CAL-A to separate the enantiomers of DIBO using detailed enzyme-substrate interaction analysis through in silico docking. Additionally, even though CAL-A is not a metalloenzyme [10], Mg^2+^ was suspected to interact with CAL-A and potential binding sites for Mg^2+^ were predicted. Finally, enantiomers of DIBO were successfully separated by CAL-A-catalyzed resolution and their absolute configurations were determined with X-ray diffraction (XRD) analysis.

## 2. Results and Discussion

Selection of enzyme and solvent: Altogether, 18 hydrolases, mostly lipases (listed in the Materials and Methods section), were screened for the *O*-acylation of DIBO (*rac*-**1**; 5.0 mM) with vinyl acetate (150 mM; R^1^ = Me, R^2^ = CH=CH_2_; Scheme 1) in methyl *tert*-butyl ether (MTBE). The preliminary reaction conditions were based on our earlier works and optimization studies with CAL-A [6,8]. Most of the evaluated hydrolases did not accept DIBO as a substrate. Only one lipase, CAT#NZL-101-IMB (herein CAL-A), provided the reaction with high enantiomeric ratio (*E*) of 130 and 49% conversion in 96 h. MTBE emerged as the best out of nine tested solvents as described in Supplementary Materials.

### 2.1. Enantiorecognition of DIBO by CAL-A

**Scheme 2:**
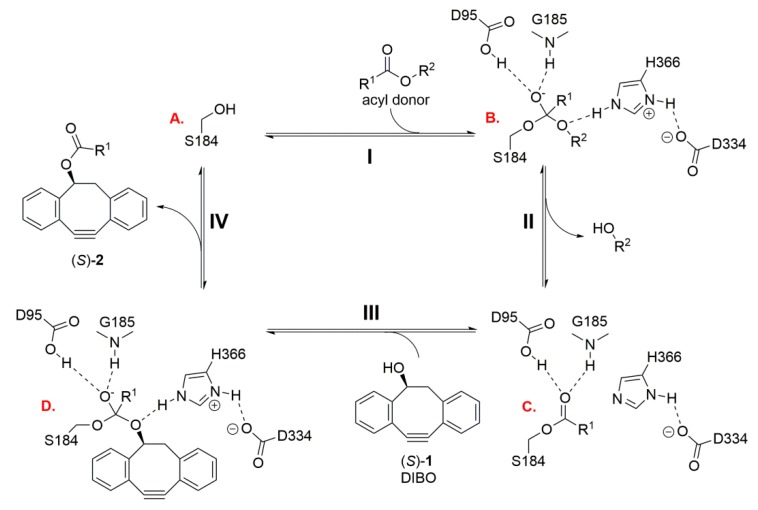
Mechanism of CAL-A-catalyzed transesterification of DIBO.

**Figure 1:**
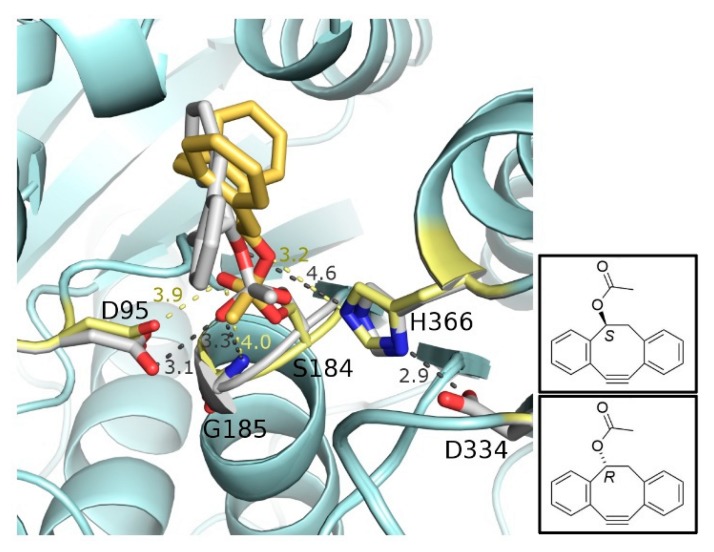
(*R*)- and (*S*)-**2** (R^1^ = Me) docked to the active site of CAL-A. (*S*)-**2** (R^1^ = Me) (yellow) likely binds to the active site of CAL-A by forming a tight interaction network of hydrogen bonds to the oxyanion hole and the catalytic residues. However, if the carbonyl oxygen of (*R*)-**2** (R^1^ = Me) (grey) is within hydrogen bonding distance to the oxyanion hole, the catalytic H366 is too far away from the other oxygen (4.6 Å) to be able to abstract the proton and form the reaction intermediate D (Scheme 2). Consequently, it is unlikely that CAL-A can form a productive reaction complex with (*R*)-**2** (R^1^ = Me). 2D structures of (*S*)- and (*R*)-**2** (R^1^ = Me) are shown to the right.

CAL-A-catalyzed *O*-acylation follows the ping-pong bi-bi mechanism, taking place in four primary steps (I-IV in Scheme 2). In steps I and II, S184 of CAL-A (A) reacts with an acyl donor giving the acyl-enzyme intermediate, Ser-OCOR^2^ (C), through formation of the first tetrahedral anionic intermediate (B). In step III, DIBO attacks the acyl-enzyme intermediate, leading to the second tetrahedral intermediate (D) and further release of the product (*S*)-**2**. To explain the ability of CAL-A to differentiate the enantiomers of DIBO, (*R*)- and (*S*)-**2** (R^1^ = Me) mimicking intermediate D, were docked to the active site of CAL-A (Figure 1). The presented results represent the best poses taking into account that, for productive binding and stabilization of the reaction intermediates, the ester carbonyl oxygen has to be within hydrogen bonding distance to the OH-group of D95 and the NH-group of G185, which together form the oxyanion hole. Moreover, the other ester oxygen has to be within hydrogen bonding distance to the catalytic H366 that works in pair with D334 [10]. (*S*)-**2** was shown to bind effectively and form a tight interaction network of hydrogen bonds. With (*R*)-**2**, by contrast, the long distance to the catalytic H366 (4.6 Å) makes H366 unlikely to abstract the proton and form the reaction intermediate D (Scheme 2), if the carbonyl oxygen is within hydrogen bonding distance to the residues in the oxyanion hole. Furthermore, the carbonyl oxygen of (*R*)-**2** slightly turns away from the oxyanion hole, thereby restricting the formation of a productive complex for catalysis. Several polar residues within 4 Å of the acetyl group are also contributing to the destabilizing effect on the complex formation. Consequently, it is unlikely that CAL-A can form a productive reaction complex with (*R*)-**2** and, hence, is highly stereoselective for the *S* enantiomer of DIBO.

### 2.2. In Silico Docking of Acyl Donors

**Figure 2:**
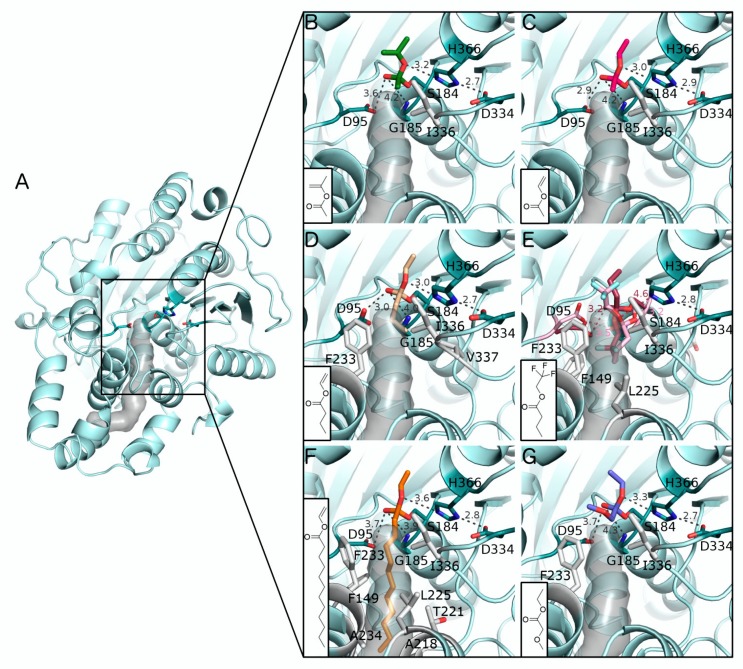
Predicted binding modes of the investigated acyl donors. (**A**) The flap region (residues D425–P441) was removed from the structure of CAL-A (PDB ID: 3GUU [18]) to mimic the interfacially activated substrate-binding conformation with the catalytic amino acids and amino acids forming the oxyanion hole exposed (shown as sticks). CAL-A also has an acyl binding pocket extending from the active site into the lid domain (gray surface). The catalytic amino acids and the oxyanion hole are shown as sticks in dark turquoise, their hydrogen bonds (with distances) to the ligands in gray dashes, amino acids within 4 Å of the ligands as gray sticks, and the acyl binding pocket as a gray surface to show the positioning of the ligand in relation to it. Predicted binding modes of (**B**) isopropenyl acetate (dark green sticks), (**C**) vinyl acetate (pink sticks), (**D**) vinyl butanoate (wheat sticks), (**E**) trifluoroethyl butanoate (two possible binding modes represented in light and dark pink sticks), (**F**) vinyl laurate (orange sticks), and (**G**) ethyl methoxyacetate (light purple). 2D structures of the acyl donors are included as inserts.

To accommodate an acyl donor, CAL-A has a narrow and hydrophobic acyl binding pocket extending into its lid domain, while the active site itself is covered by a flap that moves away during interfacial activation to expose the binding pocket. The pocket is able to accommodate acyl residues with an approximate maximum of 20–25 methylene units [10]. It has been shown that the commercially available CAL-A-preparation contains also a truncated enzyme variant lacking the active-site flap. Both forms of CAL-A have been shown to be catalytically active and catalyze the acylation of alcohols similarly with the same enantioselectivity [11]. This provides, accordingly, a good basis for the docking simulations where the flap region of CAL-A was removed to mimic the active conformation. This conformation was able to accommodate all acyl donors in the docking simulations (Figure 2A). The examined acyl donors were isopropenyl acetate, vinyl acetate, ethyl acetate, vinyl butanoate, trifluoroethyl butanoate, vinyl laurate, and ethyl methoxyacetate. To analyze the interactions of these acyl donors with CAL-A, the donors were covalently docked to the catalytic S184 (intermediate B in Scheme 2) and the chosen poses represent complexes where the requirements for productive binding and stabilization of the reaction intermediates listed above are satisfied.

The docked poses of both isopropenyl acetate (Figure 2B) and vinyl acetate (Figure 2C) to the CAL-A active site indicate that they can form the crucial hydrogen bonding network described above, which is essential for enzymatic activity. In addition, their interactions with CAL-A are mainly formed with the active site, without additional interactions with the acyl binding pocket, except for I336 at the entrance of the pocket. This explains the excellent reactivity seen in the experimental results (Table 1, entries 1 and 2). As an exception among acetates (entries 1-3), the reactions were slow with ethyl acetate (entry 3), as also previously observed [16]. Compared to vinyl and isopropenyl acetates, ethyl acetate involves a weaker leaving group and is a less activated ester as well [17]. This likely causes the slow reaction, since the docked pose of ethyl acetate is identical to the one for vinyl acetate (Figure 2C), and addition of isopropenyl acetate into reaction mixture resulted in a rapid formation of the acetate (*S*)-**2**. 

Interestingly, the reactions with vinyl and trifluoroethyl butanoate tended to stop at ca. 15% conversion in MTBE (entries 4 and 5). A similar phenomenon was seen in the reaction of long-chain vinyl laurate, where the reaction stopped at 7% conversion (entry 6). Inhibition was the most plausible explanation and it was verified by adding isopropenyl acetate into the retarded reactions. Because the transformation from *rac*-**1** into its acetate product occurred only at negligible rate, it could be interpreted that the enzyme activity had been decreased by inhibition. The docking results of vinyl butanoate, trifluoroethyl butanoate, and vinyl laurate are presented in Figure 2D–F. Contrary to isopropenyl and vinyl acetates, these acyl donors also interact with the acyl binding pocket of CAL-A. They form hydrophobic interactions with several residues: F233, I336, and V337 (vinyl butanoate); F233 and I336 (trifluoroethyl butanoate); and F149, A218, T221, L225, F233, A234, and I336 (vinyl laurate). Moreover, the binding predictions of trifluoroethyl butanoate (Figure 2E) resulted in two equally possible conformations; one with the trifluoroethyl group pointing towards the solvent and another with this group pointing towards the acyl binding pocket. Either of these conformations would enable interactions with the acyl binding pocket. 

**Figure 3:**
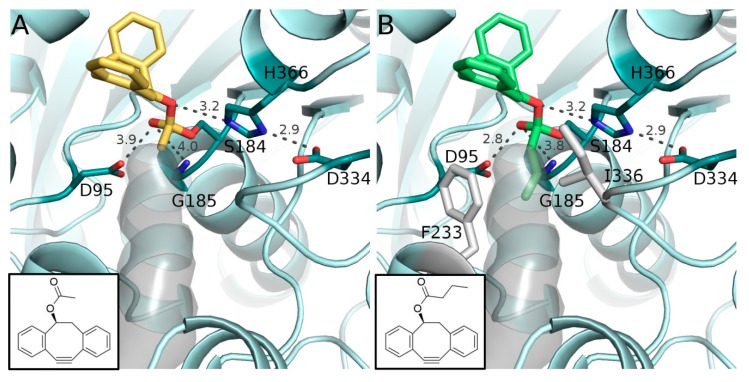
Predicted binding mode of DIBO to CAL-A. The docked complexes represent the second acyl-enzyme intermediates (D, Scheme 2) and indicate that both acetylated (yellow sticks, (**A**)) and butanoylated DIBO (green sticks, (**B**)) can bind to the catalytic amino acids and the amino acids of the oxyanion hole (dark turquoise sticks). However, besides these hydrogen bonds, butanoylated DIBO can interact with residues F233 and I336 (gray sticks), which are located within 4 Å distance from the hydrocarbon chain of the propyl group. These hydrophobic interactions likely increase the binding affinity, causing the slower release of the product. 2D structures of the two DIBO structures are included as inserts.

However, hydrophobic interactions between acyl donors and CAL-A are unlikely to fully explain the observed inhibition since butanoates have successfully been used in many kinetic resolutions [6,8], inhibition being reported in only one paper [19]. This indicates that the substrate, DIBO, must participate in the inhibition. The second tetrahedral intermediate (D) formed in step III (Scheme 2) was considered a likely inhibitor of the enzyme. Docking simulations were performed to compare the binding properties of acetylated DIBO ((*S*)-**2**, R^1^ = Me, Figure 3A) and butanoylated DIBO ((*S*)-**2**, R^1^ = Pr, Figure 3B). In these, intermediate D (R^1^ = Me) was found to bind to the catalytic amino acids and to the amino acids in the oxyanion hole through a binding mode that satisfies all requirements for productive binding and stabilization of the reaction intermediate. Besides these hydrogen bonds, intermediate D (R^1^ = Me) does not reach the acyl binding pocket of CAL-A and makes no additional interactions with the hydrophobic amino acids lining this pocket. Similarly, intermediate D (R^1^ = Pr) satisfies the same hydrogen bond requirements to the catalytic amino acids and to the amino acids of the oxyanion hole. Most importantly, the hydrocarbon chain of the propyl group is long enough to form hydrophobic interactions with F233 and I336 at the entrance of the acyl binding pocket. These interactions likely increase binding affinity and attenuate product release, leading to enzyme inhibition.

Finally, with the last investigated acyl donor, ethyl methoxyacetate, no reaction was detected (entry 7, Table 1). In this case, inhibition was not present since addition of isopropenyl acetate into the reaction mixture (*c* = 0%) gave 41% of (*S*)-**2** (R^1^ = Me) after the subsequent 96 h. Hence, ethyl methoxyacetate was not accepted as a substrate for CAL-A. The docking results for ethyl methoxyacetate show poses that remain on the surface of the enzyme without forming interactions inside the acyl binding pocket (Figure 2G). This is likely due to the hydrophilicity of the molecule, which makes it unlikely to bind to CAL-A since, contrary to the other acyl donors, the acyl binding pocket repels the hydrophilic chain, leaving a part of the molecule exposed to the solvent. Even though the smaller acyl donors do not interact with the acyl binding pocket because of the short chain, they differ from ethyl methoxyacetate as the whole molecule can be accommodated and stabilized in the active site. This is in line with the experimental results, showing no reaction with this acyl donor.

### 2.3. Effect of Drying Agents and Magnesium Ions

As a distinct feature of the CAL-A-catalyzed *O*-acylation of DIBO, ee_s_ increased very slowly after 24 h. With isopropenyl acetate, increase was smooth (entries 1 and 2, Table 2), whereas with vinyl acetate, ee_s_ did not increase after reaching 91% (entry 7). The presence of water, allowing hydrolysis of (*S*)-**2** (R^1^ = Me), is the most common reason for this [9]. Accordingly, even though hydrolysis of the formed reaction product is a common problem in enzymatic *O*-acylations, a certain amount of water is necessary for the proper functioning of the enzyme. The resolution reaction, however, does not produce water into the reaction medium, but possible sources of water include the reactants, the solvent, the catalyst and the atmosphere. To maintain an optimal water content in the reaction medium, molecular sieves [20] and pairs of salts, as the anhydrous and hydrated forms [21], have been successfully used. In this study, molecular sieves and anhydrous Mg^2+^ salts were added into the reaction mixtures to bind water and to prevent the hydrolysis of (*S*)-**2** (R^1^ = Me) into (*S*)-**1**.

In the presence of isopropenyl acetate, these drying agents, with only minor effects on enantioselectivity, enabled reaching 93–97% ee_s_ in 24 h, in contrast to 90% ee_s_ in their absence (entries 1, 3–5, Table 2). With vinyl acetate, use of anhydrous MgCl_2_ clearly enabled the reaction to proceed further, where 96% ee_s_ was reached in 24 h (entries 7 and 8).

Surprisingly, the hydrated salt MgCl_2_·6H_2_O also had a strong impact on the reaction. MgCl_2_·6H_2_O already contains a maximum number of water molecules and is thus unable to bind water from the reaction medium. Therefore, the differences detected in the reaction rates or enantioselectivities suggest some other explanation than binding of water from the reaction medium. With vinyl acetate, 96% ee_s_ was reached in 24 h also in the presence of MgCl_2_·6H_2_O (entry 9), whereas in its absence, increase in ee_s_ tended to stop at 91% (entry 7). With isopropenyl acetate, the reaction rate just slightly decreased, whereas enantioselectivity increased from 240 to 355 as expressed by the *E*-values (entries 1 and 6).

**Figure 4:**
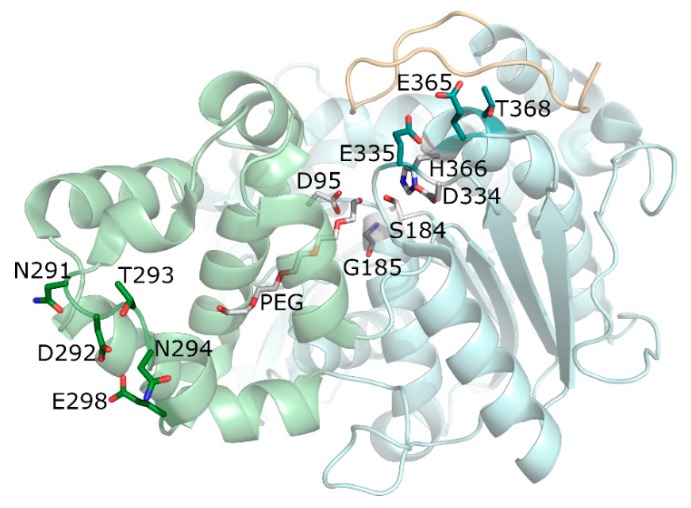
Predicted Mg^2+^ binding sites. Mg^2+^ has a positive effect on the reaction rate and enantioselectivity of CAL-A. Mapping of the predicted Mg^2+^ binding sites to the CAL-A 3D structure (Protein Data Bank PDB ID: 3GUU [18]) shows two possible sites; one in the lid domain (light green) and another in the hydrolase domain (light turquoise) at the interface with the flap (wheat).

One of the available CAL-A structures (Protein Data Bank PDB ID: 2VEO [10]) contains two uranium atoms (U^4+^); one bound to the lid at the bottom of the acyl binding pocket (coordinated by D292 and E298) and another bound to the interface between the lid and the hydrolase domain (coordinated by D220 and E314). In accordance with our experimental results showing that Mg^2+^ has positive effects on the reaction rate and enantioselectivity, a prediction of Mg^2+^ binding sites in CAL-A (PDB ID: 3GUU [18]) shows that D292 and E298 in the lid also form a potential binding site for this metal (Figure 4). This is in line with the observations of Lu et al., who reported that Mg^2+^ preferentially binds to amino acid residues D and E, and that the preferred atom types surrounding the metal ion are side chain oxygens [22]. In the lid domain, this corresponds well with the predicted D292, E298, and the surrounding N291, T293, and N294. Another potential site of interest is next to D334 and H366 in the active site. E335 and E365 could coordinate the Mg^2+^ ion and the nearby T368 can contribute to a favorable Mg^2+^ binding environment with its side chain oxygen. Interestingly, this site is at the interface between the hydrolase domain and the flap, which moves away to open the active site during interfacial activation. Thus, it can be speculated that a potential Mg^2+^ binding site in this region could have an effect on the reaction rate by modulating the movement of the flap and, consequently, affect the enzyme activation and the reaction rate.

### 2.4. Separation of the Enantiomers and Determination of the Absolute Configurations

Finally, to separate the enantiomers of *rac*-**1** (DIBO), the use of MgCl_2_·6H_2_O with isopropenyl acetate seemed promising (*E* = 355), but the reaction proceeded slowly due to the presence of water, not reaching 96% ee_s_ until after 96 h. Anhydrous MgCl_2_ provided a higher reaction rate, 49% conversion and *E* of 210 (entry 5 in Table 2) in 24 h. To take advantage of the high enantioselectivity achieved with MgCl_2_·6H_2_O (entry 6), mixing of anhydrous and hydrated MgCl_2_ (1:1, *w*/*w*) led to a satisfactory compromise (ee_s_ = 91% and *E* = 230, *t* = 24 h). These conditions were then chosen for the preparative scale reaction where (*R*)-**1** (ee_s_ 95%) and (*S*)-**2** (R^1^ = Me; ee_p_ 96%) were isolated.

**Figure 5:**
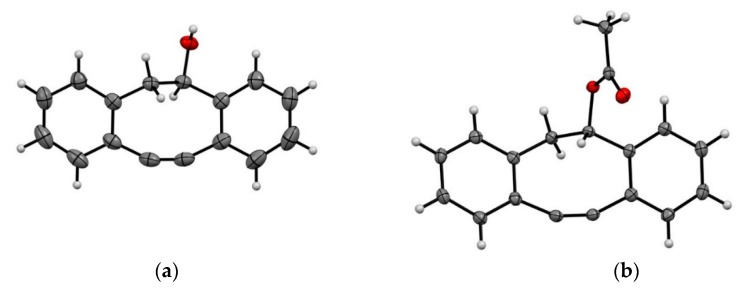
X-Ray crystal structures of (*R*)-**1** (**a**) and (*S*)-**2** (**b**) with thermal displacement parameters shown at the 50% probability level.

The single crystal X-ray crystal structures (see Supplementary Materials) of (*R*)-**1** and (*S*)-**2** are shown in Figure 5. The absolute configuration of **1** at sp^3^ HC*OH carbon was confirmed to be (*R*) with the Flack parameter value of −0.04(9) [23,24]. Although the Flack parameter for **1**, containing only one oxygen atom, had a value close to zero, the estimated standard deviation (esd) was too high for the result to be considered statistically reliable. Therefore, a single crystal of compound **2**, containing an ester group instead OH, was examined. The two oxygen atoms resulted in larger anomalous scattering than (*R*)-**1**, and the Flack parameter had a value of 0.08(4). As expected, **2** had the opposite (*S*)-configuration at sp^3^ HC*OOCCH_3_. The absolute configurations of **1** and **2**, defined as *R* and *S*, respectively, are in accordance with the molecular modeling estimations described in Figure 1.

## 3. Materials and Methods

### 3.1. General Information

The enzymes screened for the *O*-acylation of DIBO (*rac*-**1**): four different preparations of lipase A from *Candida antarctica*: NZL-101-IMB (BioCatalytics), Immozyme CALA-T2-150 (ChiralVision), VZ-1031-3 (Viazym), and CAL-A (20%) on Celite [15]. Lipase B from *Candida antarctica* (Novozym 435, Novozymes), lipase from *Burkholderia cepacia* (20% on Celite [15], Amano), *Rhizomucor miehei* (Lipozyme RM IM, Novozymes and Immozyme IMMRML-T2-150, ChiralVision), *Thermomyces lanuginosus* (Lipozyme TL IM, Novozymes and NZL-105-LYO, Biocatalytics), *Pseudomonas fluorescens* (ChiralVision), *Rhizopus oryzae* (ChiralVision), *Candida rugosa* (ChiralVision), porcine pancreas (Sigma), and *Penicillium roquefortii* (20% on Celite) [15]. Acylase I from *Aspergillus* (Fluka), alcalase from *Bacillus licheniformis* (Sigma, Steinheim, Germany), and esterase from porcine liver (Fluka). Accordingly, three enzymes were immobilized by adsorption on Celite [15]. All other enzymes were commercially available in immobilized form, or they were used as such without immobilization.

The substrate *rac*-**1** (1027338-06-2, ≥95%) was acquired from WuXi AppTec. MTBE, DIPE, 2-methyltetrahydrofuran, acetonitrile, diethyl ether, toluene, hexane, and propan-2-ol were purchased from Sigma-Aldrich. 1,2-Dimethoxyethane, vinyl acetate, isopropenyl acetate, vinyl butanoate, and ethyl methoxyacetate were products of Aldrich. 2-methylbutan-2-ol and vinyl laurate were obtained from Fluka and petroleum ether from J.T.Baker. 2,2,2-Trifluoroethyl butanoate was prepared from butanoyl chloride and trifluoroethanol. Solvents and acyl donors were dried with molecular sieves (3Å, Sigma-Aldrich) to minimize their water content. Salts used in the enzymatic reactions were anhydrous MgCl_2_, MgCl_2_·6H_2_O (both from Merck, Darmstadt, Germany), and MgSO_4_ (J.T.Baker, Deventer, Holland).

### 3.2. Instrumentation

Enzymatic reactions were monitored with an Agilent 1100 HPLC-UV (λ = 305 nm) instrument equipped with a Chiralcel^®^ OD-H column (4.6 × 250 mm, 5 µm, Chiral Technologies Europe, Illkirch, France). A detailed description of the procedure is available in the Supplementary Materials.

NMR spectra were determined using a Bruker 500 MHz AVANCE III NMR spectrometer (Bruker, Rheinstetten, Germany). The solvent was CDCl_3_. The observed ^1^H and ^13^C NMR spectra are reported in Supplementary Materials.

Single-crystal X-ray data were acquired using a Rigaku SuperNova dual-source Oxford diffractometer equipped with an Atlas detector (Agilent XRD, Oxford, United Kingdom), using mirror-monochromated Cu-*K*α (λ = 1.54184 Å) radiation.

Exact masses were measured with a Bruker Daltonics micrOTOF high-resolution mass spectrometer (Bruker, Bremen, Germany).

### 3.3. Analytical Scale Reactions

In a typical analytical scale reaction, *rac*-**1** (5.0 mmol/L) in a solvent (1.00 mL) was pipetted into the reaction vial containing the enzyme preparation (60 mg/mL). The reaction was started by adding the acyl donor (150 mM). Lipase-catalyzed reactions were carried out at room temperature and ambient pressure. A reciprocating platform shaker (Heidolph Promax 2020, speed 170 1/min) was used to mix the solution. Samples (0,1 mL) were taken at intervals by filtering off the enzyme. Conversions of the reactions were determined with HPLC analyses (Agilent, Santa Clara, USA). Determination of enantiomeric ratio (*E*) was based on the equation *E* = {ln[(1 − *c*)(1 − ee_s_)]/ln[(1 − *c*)(1 + ee_s_)]}, which relates the conversion of the racemic substrate (*c*) and enantiomeric excess of the substrate and the product, where *c* = ee_S_/(ee_S_+ee_P_) [25]. Several samples were collected during the reactions and *E* was achieved as the slope of the linear line ln[(1 − *c*)(1 − ee_S_)] versus ln[(1 − *c*)(1 + ee_S_)]. To reduce the calculation error caused by hydrolysis of (*S*)-**2**, *E* values have been calculated on the basis of several samples at an early stage of the reactions where hydrolysis of the product would be expected to be negligible.

### 3.4. Preparative Scale O-acylation of Rac-1

*Rac*-**1** (R^1^ = Me; 0.75 g, 3.4 mmol) was dissolved into MTBE (136 mL). Addition of CAL-A (CAT#NZL-101-IMB; 13.6 g), anhydrous, and hydrated MgCl_2_ (both 9.1 g) and isopropenyl acetate (11.3 mL, 102.2 mmol) started the reaction. Lipase-catalyzed reactions were carried out at room temperature and ambient pressure. A reciprocating platform shaker (Heidolph Promax 2020, speed 170 1/min) was used to mix the solution. The reaction was stopped at 50% conversion (*t* = 48 h) by filtering off the enzyme.

(*R*)-**1** and (*S*)-**2** were separated by column chromatography on silica gel using petroleum ether:acetone (95:5 and later 1:1, *v*/*v*) as eluent. During the elution, fast and qualitative silica gel thin-layer chromatography (TLC) was applied to follow the fractionation. Spots were detected under UV illumination (Camag, Muttenz, Switzerland). Finally, fractions were combined and evaporated with a rotary evaporator, followed by drying in a desiccator.

## 4. Conclusions

Racemic 4-dibenzocyclooctynol (DIBO, *rac*-**1**) has been previously introduced as part of a potential PET imaging agent to diagnose pancreatic cancer. Instead of racemates, enantiopure imaging agents are preferred to ensure specific biological target binding and, additionally, to reduce the radiation dose of patients. In this paper, we introduce a novel biocatalytic approach to separate the enantiomers of a highly strained and bulky molecular species, DIBO, the enantiomers of which have been difficult to isolate or synthesize with asymmetric catalysts. Kinetic resolution was performed with high enantioselectivity (*E* = 230) by lipase A from *Candida antarctica* (CAL-A). In optimized conditions, (*R*)-**1** (ee 95%) and (*S*)-**2** (R^1^ = Me; ee 96%) were separated using methyl *tert*-butyl ether as the solvent and isopropenyl acetate as the acyl donor. Accordingly, both enantiomers of DIBO became simultaneously available for biological experiments. The current research results exemplify the unparalleled ability of CAL-A to accommodate bulky substrate molecules by opening of the flap during interfacial activation and subsequently to provide practically unlimited space at the active site. The basis of the ability of CAL-A to distinguish the DIBO enantiomers was further shown to reside in the hydrogen bonding network of the catalytic machinery, where the tetrahedral anionic intermediate leading to (*R*)-**2** is not able to form a hydrogen bond with the catalytic H366 while the carbonyl oxygen is pointing slightly away from the oxyanion hole.

In silico docking was used to explain and illustrate the binding properties of several acyl donors. The results are in excellent accordance with the obtained experimental data. The acyl binding pocket is highly hydrophobic, and the long hydrocarbon chains of butanoate and laurate were shown to bind tightly into the acyl binding pocket with dispersion forces. This likely results in slower reaction rate and even inhibition of CAL-A. Thus, short-chain vinyl and isopropenyl acetates were clearly the best acyl donors, whereas methoxyacetate with a polar chain was repelled by the acyl binding pocket of CAL-A.

Anhydrous magnesium salts were successfully used for binding water, thus preventing the hydrolysis of the product (*S*)-**2** into the starting material (*S*)-**1**. In preparative scale resolution, a 1:1 (*w*/*w*) mixture of anhydrous and hydrated MgCl_2_ was the best compromise to achieve high enantioselectivity and a sufficiently rapid reaction. Surprisingly, fully hydrated Mg^2+^ hexahydrate alone impacted positively both the reaction rate and the enantioselectivity, which suggests, as one option, binding of Mg^2+^ to the enzyme during the catalytic process. Two possible binding sites for Mg^2+^ were predicted that could potentially explain the experimentally observed effects on the reaction rate. The exact coordination sites and effects of other metal ions remain to be investigated in future studies. Another essential future target is to carry out in vivo PET studies with imaging agents containing enantiopure DIBO in order to determine their biological targeting characteristics.

## Figures and Tables

**Table 1:** CAL-A-catalyzed (NZL-101-IMB; 60 mg/mL) *O*-acylation of *rac*-**1** (5.0 mM) with various acyl donors (150 mM) (*t* = 96 h).

Entry	Acyl Donor	R^1^	R^2^	*ee_s_* (%)	*ee_p_* (%)	*c* (%)	*E* ^a)^
1	Isopropenyl acetate	Me	C(CH_3_)=CH_2_	98	96	51	240
2	Vinyl acetate	Me	CH=CH_2_	91	95	49	130
3	Ethyl acetate	Me	Et	7	>99	7	-^b)^
4	Vinyl butanoate	Pr	CH=CH_2_	18	98	15	90
5	Trifluoroethyl butanoate	Pr	CH_2_CF_3_	19	>99	16	>200
6	Vinyl laurate	CH_3_(CH_2_)_10_	CH=CH_2_	8	96	7	-^b)^
7	Ethyl methoxyacetate	MeOCH_2_	Et	0	-	0	-

^a)^ Enantiomeric ratio. ^b)^ *E* not determined because of the early stage of the reaction.

**Table 2:** CAL-A-catalyzed (NZL-101-IMB; 60 mg/mL) acylation of *rac*-**1** (5.0 mM) with isopropenyl acetate (150 mM) in methyl *tert*-butyl ether (MTBE) in the presence of various additives (80 mg/mL).

Entry	Acyl Donor	Additives	*t* (h)	*ee_s_* (%)	*ee_p_* (%)	*c* (%)	*E*
1	Isopropenyl acetate	none	24	90	97	48	240
2	Isopropenyl acetate	none	96	98	96	51	240
3	Isopropenyl acetate	3 Å molecular sieves ^a)^	24	97	94	51	185
4	Isopropenyl acetate	MgSO_4_ (anh.)	24	95	96	50	185
5	Isopropenyl acetate	MgCl_2_ (anh.)	24	93	97	49	210
6	Isopropenyl acetate	MgCl_2_·6H_2_O	24	86	99	47	355
7	Vinyl acetate	none	96	91	95	49	130
8	Vinyl acetate	MgCl_2_ (anh.)	24	96	93	51	95
9	Vinyl acetate	MgCl_2_·6H_2_O	24	96	95	50	135

^a)^ 30 pieces, ca. 170 mg.

## Supplementary Materials

The following are available online. HPLC Analyses, Preliminary reaction conditions, Compound characteristics, Determination of absolute configurations by X-ray diffraction, Docking of acyl donors and DIBO to CAL-A and Mg^2+^ binding site prediction.
