# Supplementary material for: Candida antarctica Lipase A-Based Enantiorecognition of a Highly Strained 4-Dibenzocyclooctynol (DIBO) Used for PET Imaging
Source: Molecules. 2020 Feb 17;25(4):879. doi: 10.3390/molecules25040879 (PMC7070869; doi:10.3390/molecules25040879)
Supplement: Supplementary file 1 [file molecules-25-00879-s001.pdf]

Supporting Information

Candida antarctica lipase A-based enantiorecognition of a highly strained 4-dibenzocyclooctynol (DIBO) used for PET imaging

Saija Sirén[a,b], Käthe M. Dahlström[c], Rakesh Puttreddy[d], Kari Rissanen[d], Tiina A. Salminen[c], Mika Scheinin[e], Xiang-Guo Li[f,g]*, Arto Liljeblad[a]*

[a] Laboratory of Synthetic Drug Chemistry, Institute of Biomedicine, University of Turku, Kiinamyllynkatu 10, FI-20520 Turku, Finland
Phone: +358 50 4640 556
E-mail: artlilje@utu.fi
[b] Biochemistry, Faculty of Science and Engineering, Åbo Akademi University, Tykistökatu 6 A, FI-20520 Turku, Finland
[c] Structural Bioinformatics Laboratory, Biochemistry, Faculty of Science and Engineering, Åbo Akademi University, Tykistökatu 6 A, FI-20520 Turku, Finland
[d] University of Jyväskylä, Department of Chemistry, P.O. Box 35, FI-40014 Jyväskylä, Finland
[e] Institute of Biomedicine, University of Turku, and Unit of Clinical Pharmacology, Turku University Hospital, Kiinamyllynkatu 10, FI-20520 Turku, Finland
[f] Turku PET Centre, Åbo Akademi University and University of Turku, Kiinamyllynkatu 4-8, FI-20521 Turku, Finland
[g] Turku PET Centre, Turku University Hospital, Kiinamyllynkatu 4-8, FI-20521 Turku, Finland

Table of Contents

1. HPLC Analyses S2
2. Preliminary reaction conditions S2
3. Compound characteristics S3
4. Determination of absolute configurations by X-ray diffraction S5
5. Docking of acyl donors and DIBO to CAL-A S6
6. Mg^{2+} binding site prediction S6
7. References S7

1. HPLC Analyses

Conversions of the reactions were determined with an Agilent 1100 high-performance liquid chromatography system coupled to an ultraviolet detector (λ = 305 nm). Approximately 80 µl of reaction solution was collected with a disposable syringe and the solution was filtered through a syringe filter (Millex®-FH, 0.45 µm, hydrophobic PTFE membrane, Merck Millipore) into a HPLC vial. The sample was diluted with 1200 µl of hexane:MTBE (23:1, v/v). Separations were performed with a Chiralcel® OD-H column (4.6×250 mm, 5 µm, Chiral Technologies Europe, Illkirch, France) and an isocratic eluent composed of hexane:IPA 97:3 (v/v). Injection volume was 10 µl and column temperature was 23 °C. Retention times were 9.8, 14.6, 24.1 and 27.8 min for (*R*)-**2**, (*S*)-**2**, (*R*)-**1** and (*S*)-**1**, respectively.

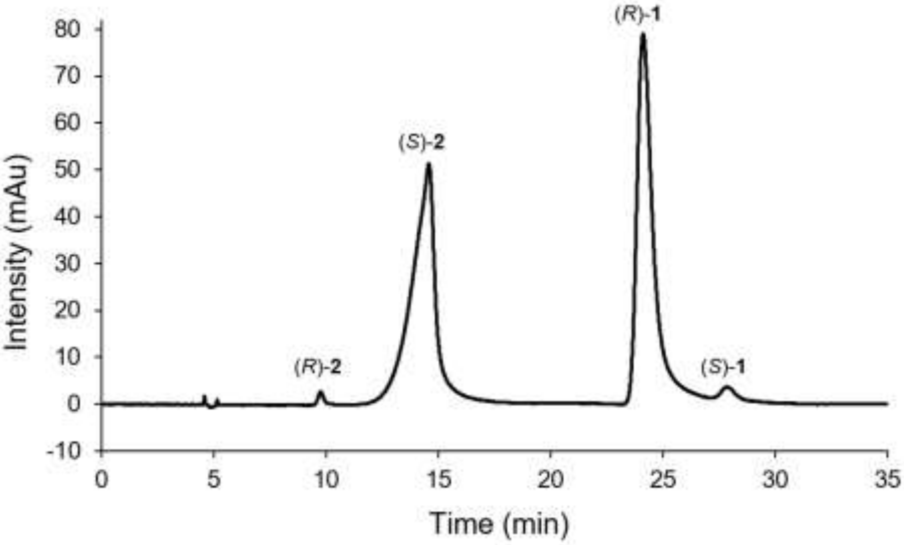

Figure S1. Chiral HPLC chromatogram of the preparative scale reaction at 24 h.

2. Preliminary reaction conditions

Nine solvents were tested in the *O*-acylation of *rac*-**1** with vinyl acetate (Table). After 96 h, the reactions in 2-methylbutan-2-ol (*tert*-amyl alcohol, ^{t}amylOH) and methyl *tert*-butyl ether (MTBE) reached close to 50% conversion (entries 1 and 2), whereas in the other solvents the reactions were clearly slower (entries 3-9). Regarding enantioselectivity, MTBE, diisopropyl ether (DIPE) and ^{t}amylOH were the best solvents with $E > 100$ (entries 1-3).

Table. CAL-A-catalyzed (NZL-101-IMB; 60 mg/mL) acylation of *rac*-**1** (5.0 mM) with vinyl acetate (150 mM, R^1 = Me, R^2 = CH=CH$_2$) in various solvents (t = 96 h).

**entry**	**solvent**	ee_s **(%)**	ee_p **(%)**	c **(%)**	E
1	^{t}amylOH	95	94	50	120
2	MTBE	92	95	49	130
3	DIPE	60	98	38	125
4	Vinyl acetate	37	98	27	70
5	2-methyltetrahydrofuran	33	98	25	90
6	Acetonitrile	25	96	21	55
7	Diethyl ether	19	98	16	60
8	Toluene	12	98	11	60
9	1,2-dimethoxyethane	10	98	10	35

3. Compound characteristics

Optical rotations were determined at 20 °C using a Perkin-Elmer Polarimeter 341. NMR spectra of the prepared enantiomers were determined using a Bruker 500 MHz AVANCE III NMR spectrometer. Exact masses were measured with a Bruker Daltonics micrOTOF high-resolution mass spectrometer.

Compound (*R*)-**1:** 0.41 g; 1.8 mmol; ee 95%; m.p. 154°C; $[\alpha]_D^{20}$ = +46.2 (c = 1.00, CH_2Cl_2).
^{1}H NMR ($CDCl_3$, 500 MHz): δ 2.92-2.95 (dd, 1H), 3.09-3.12 (dd, 1H), 4.63 (t, 1H) and 7.29-7.75 (m, 8H).
^{13}C NMR ($CDCl_3$, 500 MHz): δ 48.7, 75.3, 110.6, 112.9 and 121.2-155.6.

Compound (*S*)-**2**: 0.42 g; 1.6 mmol; ee 96%; m.p. 108°C; $[\alpha]_D^{20}$ = +87.1 (c = 1.00, CH_2Cl_2).
^{1}H NMR ($CDCl_3$, 500 MHz): δ 2.25 (s, 3H), 2.93-2.96 (dd, 1H), 3.13-3.16 (dd, 1H), 5.57 (t, 1H) and 7.29-7.51 (m, 8H).
^{13}C NMR ($CDCl_3$, 500 MHz): δ 21.2, 46.3, 76.4, 109.9, 113.0, 121.5-151.1 and 169.7.
HRMS calculated for $C_{18}H_{14}O_2$ ($[M+Na]^+$) 285.0892 and ($[M+NH_4]^+$) 280.1338. Found: 285.0896 and 280.1338, respectively.

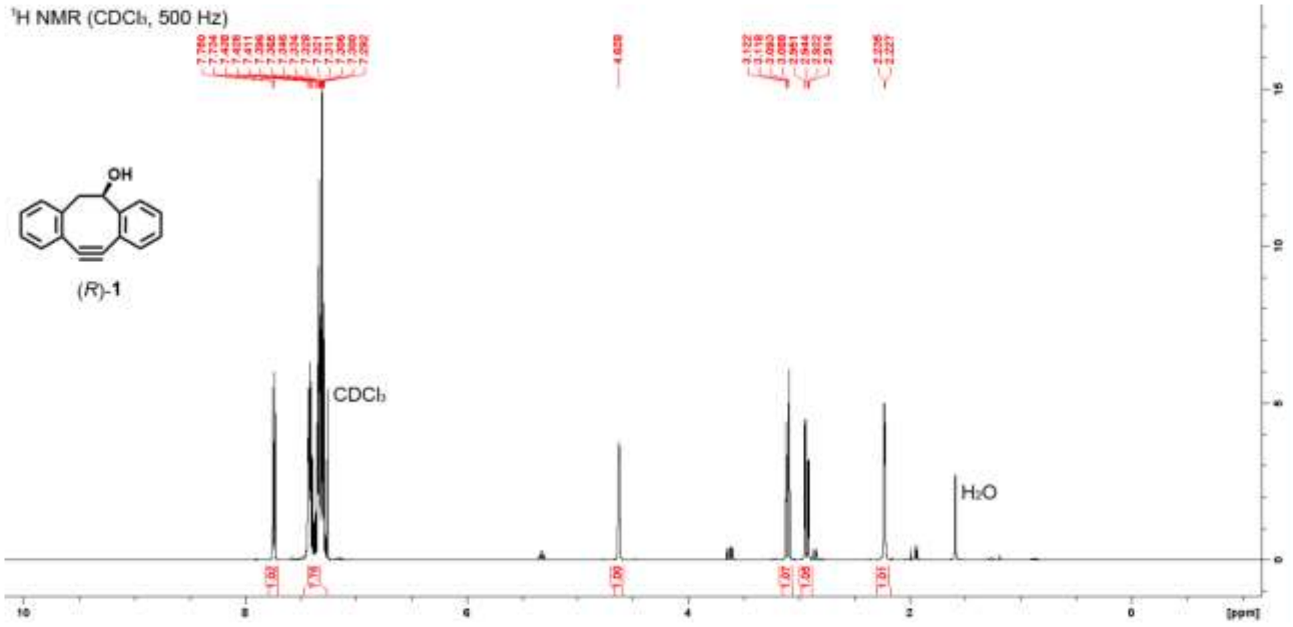

Figure S2. ^{1}H NMR ($CDCl_3$, 500 Hz) spectrum of isolated (*R*)-**1**.

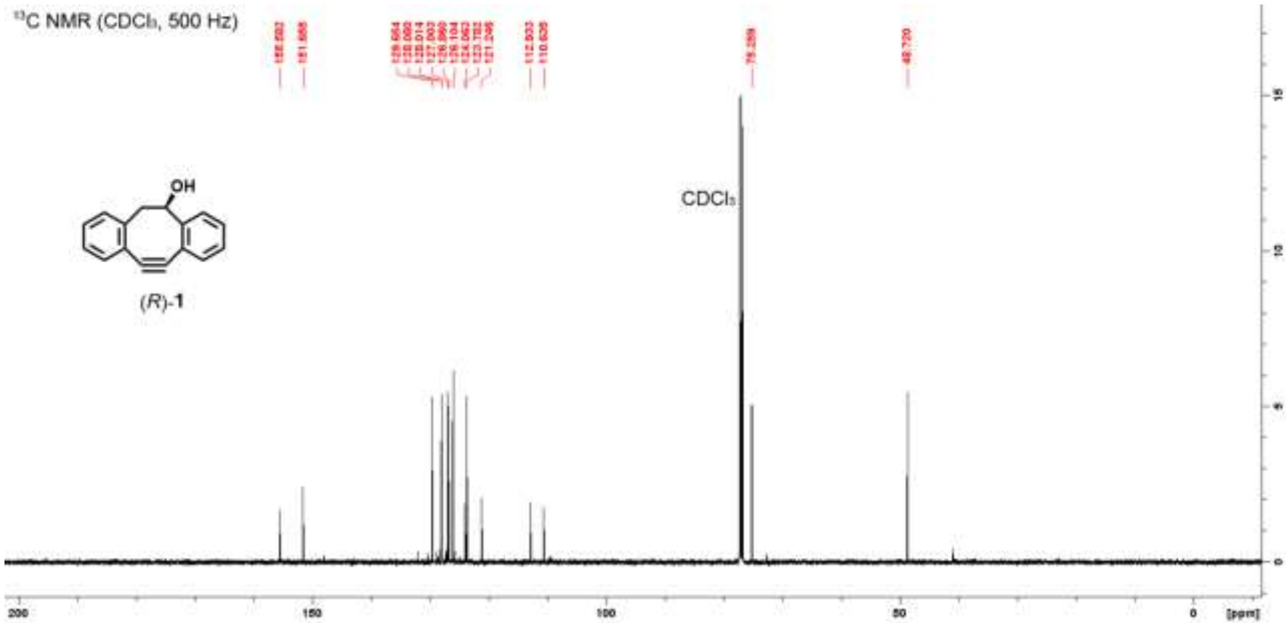

Figure S3. ^{13}C NMR ($CDCl_3$, 500 Hz) spectrum of isolated (*R*)-**1**.

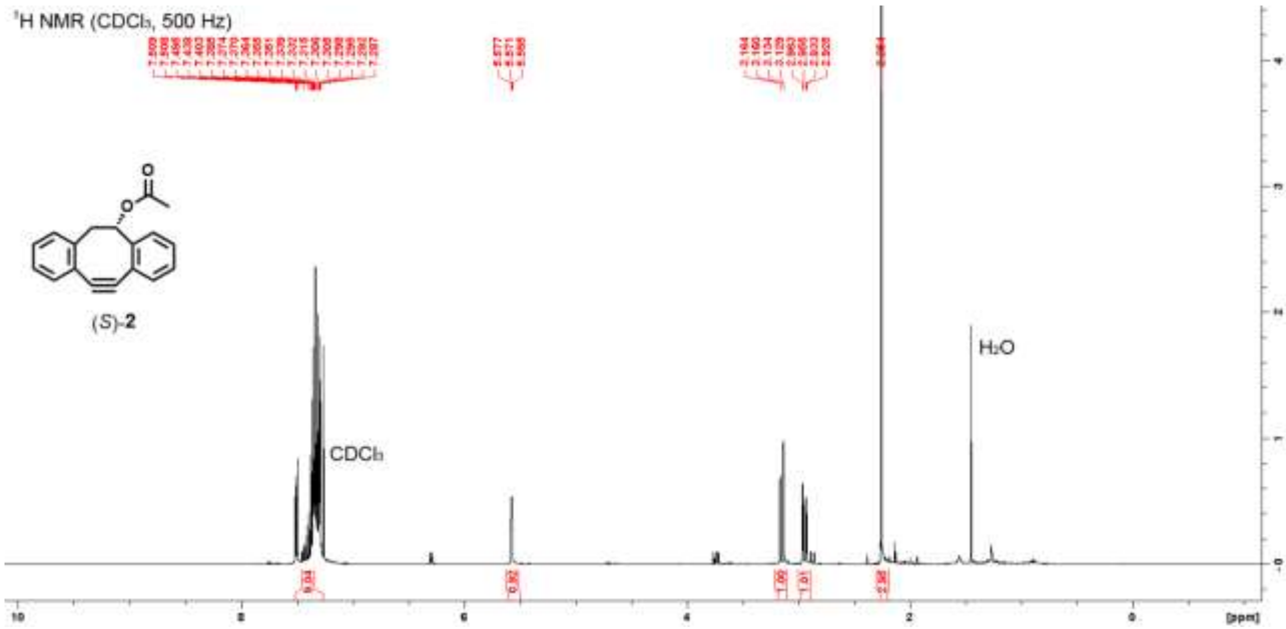

Figure S4. ^{1}H NMR ($CDCl_3$, 500 Hz) spectrum of isolated (*S*)-**2**.

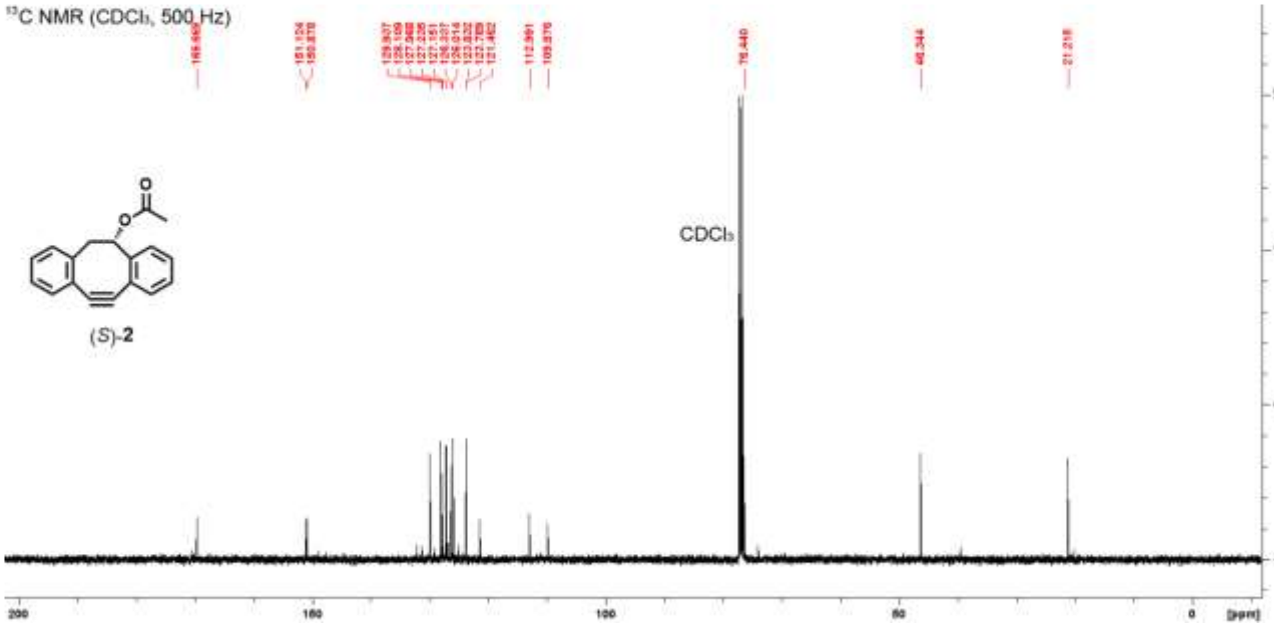

Figure S5. ^{13}C NMR ($CDCl_3$, 500 Hz) spectrum of isolated (*S*)-**2**.

4. Determination of the absolute configurations by X-ray diffraction

Chloroform was added dropwise into a solution of (*R*)-**1** or (*S*)-**2** in *n*-hexane at room temperature to dissolve the compounds. Slow evaporation of the corresponding solutions at room temperature resulted in single crystals suitable for X-ray diffraction analysis.

Single-crystal X-ray data for (*R*)-**1** and (*S*)-**2** were acquired using a Rigaku SuperNova dual-source Oxford diffractometer equipped with an Atlas detector, using mirror-monochromated Cu-*K*α (λ = 1.54184 Å) radiation. The data collection and reduction were performed using the program *CrysAlisPro*[1] and the Gaussian face index absorption correction method[1] was applied. The structure was solved with direct methods (*SHELXS*)[2] and refined by full-matrix least squares on F^2 using the *OLEX2* software[3], which utilizes the *SHELXL*-2015 module.[2]

Crystal data for (R)-**1**: CCDC-1918320, $C_{16}H_{12}O$, M = 220.26, colourless block, 0.099x0.073x0.048 mm^3, orthorhombic, space group $P2_12_12_1$, a = 4.61050(10) Å, b = 13.3571(3) Å, c = 18.7254(3) Å, α = 90°, β = 90°, γ = 90°, V = 1153.16(4) Å^3, Z = 4, Dc = 1.269 g/cm^3, F000 = 464, μ= 0.607 mm^{-1} , T = 170.01(10) K, θ_{max} = 76.793°, 23765 total reflections, 2323 with Io > 2σ(Io), R_{int} = 0.0375, 2427 data, 155 parameters, 0 restraints, GooF = 1.047, R = 0.0287and wR= 0.0726 [Io > 2σ(Io)], R = 0.0305 and wR= 0.0742 (all reflections), 0.122 <dΔρ < -0.131 e/Å^3, Flack = -0.04(9).

Crystal data for (S)-**2**: CCDC-1918321, $C_{18}H_{14}O_2$, M = 262.29, colourless block, 0.275x0.245x0.083 mm^3, orthorhombic, space group $P2_12_12_1$, a = 7.38830(10) Å, b = 9.16420(10) Å, c = 19.4997(2) Å, α = 90°, β = 90°, γ = 90°, V = 1320.28(3) Å^3, Z = 4, Dc = 1.320 g/cm^3, F000 = 552, μ= 0.677 mm^{-1} , T = 120.0(1) K, θ_{max} = 66.738°, 19434 total reflections, 2309 with Io > 2σ(Io), R_{int} = 0.0227, 2320 data, 182 parameters, 0 restraints, GooF = 1.065, R = 0.0244 and wR= 0.0651 [Io > 2σ(Io)], R = 0.0245 and wR= 0.0652 (all reflections), 0.141<dΔρ <-0.152 e/Å^3, Flack = 0.08(4).

5. Docking of acyl donors and DIBO to CAL-A

The bound PEG molecule and the flap region (residues D425 – P441) were removed from the CAL-A crystal structure (PDB ID: 3GUU[4]) to mimic the open substrate-binding conformation. Maestro (Version 11.4, Schrödinger, LLC, New York, NY, 2018) was used to draw the structures of the acyl donors and DIBO, as well as to prepare them (ligand preparation tool) and the edited protein structure (protein preparation tool) for the docking experiments. The acyl donors and DIBO were covalently docked to CAL-A S184 with Glide in Maestro, using a custom chemistry input file describing the formation of the first and second acyl-enzyme intermediates, respectively (see scheme 2 for details on the reactions). Furthermore, Mg^{2+} binding sites were predicted using the MIB: metal ion binding site prediction and docking server (cutoff for relevant Mg^{2+} binding site results is 1.8).[5,6] PyMOL (version 2.1, Schrödinger, LLC) was used to analyze the complex structures and to generate images, where the visualized pockets were calculated with Surfnet[7].

6. Mg^{2+} binding site prediction

The MIB: metal ion binding site prediction and docking server[5] was used to predict the amino acids that potentially bind Mg^{2+} (cutoff value for relevant results 1.8). The predicted binding sites in the lid and near the active site are marked in green and blue, respectively.

CAL-A residue	Score (Mg^{2+} cutoff 1.8)	CAL-A residue	Score (Mg^{2+} cutoff 1.8)	CAL-A residue	Score (Mg^{2+} cutoff 1.8)
N15	3.179	A234	2.023	K347	2.009
D18	3.179	A254	2.633	E348	2.009
D19	3.179	L256	2.633	E365	2.660
D71	2.457	R270	1.961	H366	2.179
V72	2.008	C273	1.961	A402	1.993
D100	1.961	D292	2.017	I404	1.993
D141	2.457	E298	2.017	T406	2.132
E143	2.457	D334	3.082	P407	2.132
G144	2.008	E335	3.082	L424	2.034
A158	1.921	I336	2.553	D425	2.034
L160	1.921	A343	1.967	G426	2.053
L225	2.023	V346	1.967	Q428	2.053

7. References

1. Rigaku Oxford Diffraction, 2017, CrysAlisPro Software system, version 38.46, Rigaku Corporation, Oxford, UK.
2. (a) Sheldrick, G. M. Crystal structure refinement with SHELXL. *Acta Crystallogr. Sect. C*, **2015**, *71*, 3–8. (b) Sheldrick, G. M. SHELXT–Integrated space-group and crystal-structure determination. *Acta Crystallogr. Sect. A*, **2015**, *71*, 3–8.
3. Dolomanov, O. V.; Bourhis, L. J.; Gildea, R. J.; Howard, J. A. K.; Puschmann, H. OLEX2: a complete structure solution, refinement and analysis program. *J. Appl. Crystallogr.* **2009**, *42*, 339–341.
4. Brandt, A.-M.; Li, X.-G.; Nymalm-Rejstrom, Y.; Airenne, T.; Kanerva, L.T.; Salminen, T. A. The crystal structure of lipase A from *Candida antarctica*. doi:10.2210/pdb3GUU/pdb
5. Lu, C. H.; Lin, Y. F.; Lin, J. J.; Yu, C. S. Prediction of metal ion–binding sites in proteins using the fragment transformation method. *PLoS One* **2012**, *7*, e39252.
6. Lin, Y. F; Cheng, C.W.; Shih, C. S.; Hwang, J. K.; Yu, C. S.; Lu, C. H. MIB: metal ion-binding site prediction and docking server. *J. Chem. Inf. Model.* **2016,** *56*, 2287-2291.
7. Laskowski, R. A. SURFNET: A program for visualizing molecular surfaces, cavities, and intermolecular interactions. *J. Mol. Graph.* **1995,** *13*, 323-330, 307-308.
